# Supplementary material for: Factors associating different antenatal care contacts of women: A cross-sectional analysis of Bangladesh demographic and health survey 2014 data
Source: PLoS One. 2020 Apr 29;15(4):e0232257. doi: 10.1371/journal.pone.0232257 (PMC7190106; doi:10.1371/journal.pone.0232257)
Supplement: Attachment — Submitted filename: Response to Reviewers_01-04-2020.docx [file pone.0232257.s001.docx]

Overall Comments:

We are thankful to the editor and the reviewers for their constructive comments and suggestions. We have modified the manuscript based on the comments of the editor and the reviewers, as well as the requirements of the journal. In addition, we have revised some citations and grammatical issues throughout the document. We have set up the figure and tables after the reference section.

Specific Comments:

Reviewer #1:

- The manuscript is a new study for Bangladesh where the ANC services are not yet up to desired level. To fulfil the SDGs goal 2030, this service must be improved, and the data presented in this study would be helpful to achieving the SDGs goal.

Authors response: Thank you for the complement. We hope that this study would guide the fulfillment of the SDGs goal by 2030.

- However, there are some limitations of the study since not all administrative areas are not included in this study, and the population size is too small.

Authors response: In this study, we have considered all administrative areas which were included in BDHS-2014 data collection procedure. For sample size, we have considered 17,863 women as sample. However, to avoid unnecessary and missing observation, the sample size, unintentionally, was reduced to 4,475 women. Previous studies also considered around the same size of samples, published in various renowned journals as well as PLoS ONE.

- The data are presented in tabular forms only which is very difficult to find out the correlation between the associated factors, so I suggest to have some pictorial presentation of the findings.

Authors response: We have considered to present our data into tabular forms, because both graphical and tabular forms of data presentation have similar utility.

- The authors did not consider other associated factors such as mode of delivery, abortion, and other peri-parturient diseases etc.

Authors response: We have considered the factors based on previous literature and all these studies did not consider the above-mentioned factors. So, we did not consider these variables as well. We are expecting future study would address the aforementioned factors with ANC contact.

- The teenager mothers are more involved as well interested in mass media, however, ANC service in this group of women are less than elder groups which is a contradictory issue, and not discussed well.

Authors response: Our study did not address the association between maternal age and media exposure. Moreover, our findings suggest that older women have greater ANC contact than the teenage mothers, and explained the association in the discussion (It is apparent that unlike teenage mothers, older women often contacted ANC services in Bangladesh, and the findings is aligned with previous studies. Some studies suggest that teenage mothers are less likely to seek ANC service [45], mainly due to the fear of social stigma [46]).

- Check the reference number 30, and correct the journal name. Others are okay.

Authors response: We have updated the references as per the guideline of the journal as well as your suggestion.

Reviewer #2:

Specific comments are as below:

- Abstract: I would suggest to consider changing word ‘incidence’ when it was used to ‘78.4% women had the incidence of ANC contacts’ and many other occasions as it was inappropriately used in this cross-sectional survey-based article.

Authors response: We have modified the manuscript considering your suggestion.

- Last sentence in Abstract is overly complex, split this in smaller sentences to make issues more explicit.

Authors response: We have split the last sentence into three separate, but complementary sentences.

- 1st sentence in 2nd paragraph started with ‘NC has been defined … check what it is NC stands for…

Authors response: We have made the correction by replacing “NC” by “ANC” as per your suggestion.

- 4th paragraph in Introduction ‘Only a handful ** were conducted to find out the association between determinants and contents of ANC contacts in Bangladesh [11, 19]. All previous studies have used data from demographic and health survey: so, what?’ Pls check and restate.

Authors response: The sentence, you’ve mentioned, has been removed and the previous sentence was restructured with necessary changes.

- Last sentence in Introduction is unnecessary here.

Authors response: We have removed the last sentence in the introduction section, based on your instruction.

- Pls restate the tables 2-4 heading as started as ‘Association …….

Authors response: we have restated the headings of the Table 2-4 with necessary modifications.

- All the tables are placed continuously in one setting, mix them up along with the relevant texts.

Authors response: Now we have placed all the tables consecutively at the end of the article.

- Pls consistently use 2 digits after decimal all over.

Authors response: Based on the published article in PloS One journal and your suggestions, we have rearranged this table by using 2 digits after the decimal.

- In several occasions sub-heading started with ERROR! Pls check.

Authors response: Thank you. We have checked and updated our paper.

- Have it read by a senior author?

Authors response: yes.
